# Global patterns of human and livestock respiration

**DOI:** 10.1038/s41598-018-27631-7

**Published:** 2018-06-18

**Authors:** Qixiang Cai, Xiaodong Yan, Yafei Li, Leibin Wang

**Affiliations:** State Key Laboratory of Earth Surface Processes and Resource Ecology, Faculty of Geographical Science, Beijing Normal University, Beijing, 100875 China

## Abstract

Carbon emissions from human and animals has been neglected by previous studies in estimating the carbon cycle of ecosystem. This study first estimates the spatial-temporal patterns of carbon emissions density from human and livestock respiration among countries around the world from 1960–2014. Then we simulate the soil heterotrophic respiration (R_h_) to analyze the contribution of human and livestock respiration to total heterotrophic respiration of global ecosystem. Our results show that the respiration of human and livestock respectively contribute more than 1% of the total carbon output from heterotrophic respiration in most countries and affect more than 5% in almost half of the countries. Moreover, the effect of livestock respiration is slightly greater than that of human beings. Therefore, the estimation of heterotrophic respiration should not only consider R_h_ in these countries, human and livestock respiration are equally important in the research on regional carbon budget.

## Introduction

The atmospheric carbon dioxide input-output balance could be expressed by the source and sink of CO_2_. The source caused by respiration mainly includes the autotrophic respiration of terrestrial plants and the heterotrophic respiration of terrestrial ecosystems^1^. Studies show that the global autotrophic respiration of plants is about 60Pg C yr^−1^, which is approximately equal to the net primary production (NPP), and the decomposition of soil and litter is about 50 Pg C yr^−1^ ^2^. Therefore, the respiration is an important process of terrestrial ecosystem carbon cycle, while heterotrophic respiration in terrestrial ecosystems including breathing of fungi, bacteria, microbes and fauna in soil, as well as respiration from aboveground animals and human beings. However, previous studies on respiration in terrestrial ecosystem primarily focused on the soil respiration (R_S_) in ecosystems such as forests, farmlands, grasslands^3–5^. Moreover, they usually ignored the respiration of human and animals due to a relatively small proportion compared with soil respiration on a global or regional scale.

Nevertheless, studies also suggested that the impact of population density on carbon emissions and carbon balance in urban as well as its surrounding areas have regional differences^6^. As for the animals, grazing is usually used as a land management practice to represent actual land use in some models of grassland soil organic matter^7^. Therefore, it should be noticed that human and animals participate in carbon cycle by taking food or breathing for energy metabolism. Currently, the calculation of respiration of human and aboveground animals is more common in the analysis of carbon balance within urban system. For example, an earlier study by Koerner (2002) from Arizona State University show that human respiration produce almost 1.6% input of total CO_2_, while soil respiration contribute 15.8% of the total annual CO_2_ emissions^8^. It means human respiration play a significant role in this study case. An urban metabolism approach and relevant emission processes was simulated in the Sunset community in Vancouver, Canada. The result show that out of all carbon emissions, 8% (0.39 Kg C m^−2^ yr^−1^) are from human respiration, compared with 5% (0.33 Kg C m^−2^ yr^−1^) from respiration of soil and vegetation^9^. Both of those researches turn out a great proportion of carbon emissions from human respiration, compared with that of soil respiration. In addition, carbon cycle of urban system in the city of Nanjing, China was also studied with the consideration of large livestock such as pigs and cattle, and the vertical carbon output from livestock is a third to a half of that from human respiration^10^. As the previous study mentioned above, researches on carbon cycle in ecosystem have mostly neglected the respiration of the aboveground creatures, while humans and animals (especially livestock) respiration were more common in urban systems.

On another hand, as an indicator of temperature sensitivity of respiration, a *Q*_10_ value around 1.4 across climate zones and ecosystem types have been estimated in a study at global ecosystem level^11^. Similarly, human and animals breathing in a certain temperature range would increase with the temperature rising^12–14^. Some researchers evaluated the temperature sensitivity (*Q*_10_) of metabolic rate of mammals as 2.4 or higher^15–17^. At the same time, human activities such as sports will cause an increasing intensity of breathing in a period, because oxygen uptake in exercise period is almost 5 times larger than that in recovery period^18^. Therefore, under the background of climate warming, carbon emissions from human and animals respiration would be greater than basal metabolic rate (BMR) calculated in this study.

Here we calculate the carbon emissions from human and livestock in different countries among the period of 1961–2014 and analyze the global pattern of human and livestock respiration. Then we estimate the global R_h_ and discuss their contributions to total respiration in ecosystems. This research focus on the value and contribution of each component of heterotrophic respiration (human respiration, livestock respiration and Soil heterotrophic respiration) in administrative regions, and to discuss whether the contribution of respiration from human and animals has a significant impact on the ecosystems in our study area. Additionally, considering the effects of population gathering and livestock breeding caused by urbanization, human and livestock respiration might have different degree of impact on the estimation of regional carbon emissions.

## Results

### Individual respiratory carbon emissions

We collected the data of surface areas, population and livestock production for estimating human and animals respiration. The carbon emissions from per person or each livestock was calculated by the amount of oxygen consumption according to the basal metabolic rate (BMR)^19–22^ (see Supplementary Table S1). The parameters of carbon emission per unit individual as well as the species are listed in Table 1. The metabolic enhancement caused by ambient temperature, exercise metabolism and other factors was neglected, and BMR was the only factor to estimate the individual respiratory carbon emission. We emphasize that all the animal species involved in this study are domestic animals (see Methods).

**Table 1:** Data sources and main parameters used for the calculation of carbon emissions from human and livestock.

Individual respiratory carbon emissions (kg C yr^−1^)	Species*	Data Source
217.00	Cattle	Food and agriculture organization of the United Nations (FAO)
	Buffaloes	
47.87	Sheep	
32.10	Goats	
19.36	Pigs	
1.25	Chickens	
	Ducks	
	Geese and guinea fowls	
	Turkeys	
	Pigeons, other birds	
305.04	Horses	
	Asses	
	Mules	
191.00	Camels	
7.04	Rabbits and hares	
1.50	Rodents, other	
191.00	Camelids, other	
68.99	Human being	The World Bank

*We unified the units of parameters into each year, and assumed all kinds of poultries as broiler. Therefore, we supposed that the life span of each poultry was 45 days and the life span of each pig was half a year. As it is hard to distinguish the economic use of sheep and goat, the slaughter age of meat-producing sheep and goats was not taken into consideration. We assumed all the species except poultries and pigs can live for more than one year.

### Carbon emission from human and livestock respiration

**Figure 1:**
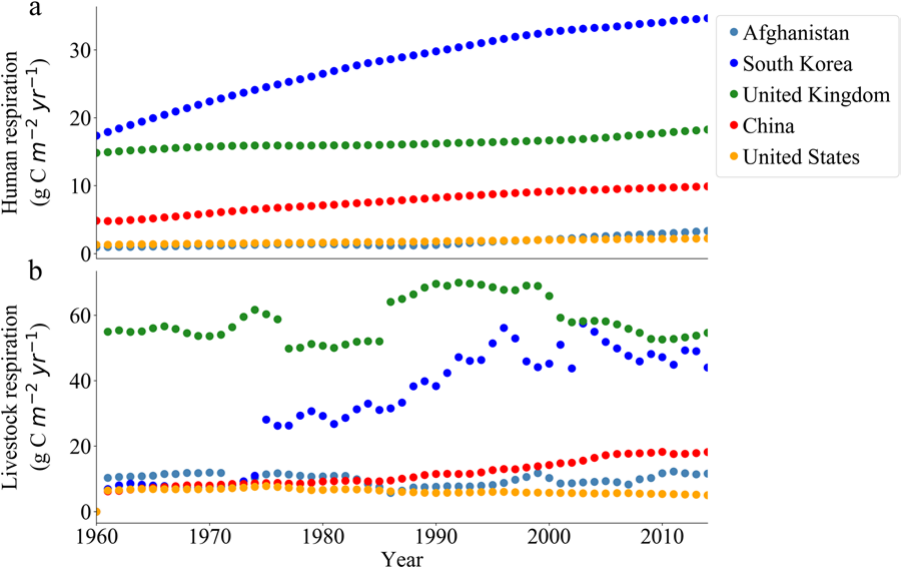
Variation of annual carbon emissions (g C m^−2^ yr^−1^) from (**a**) human and **(b)** livestock respiration in Afghanistan, South Korea, United Kingdom, China and United States. The figure was generated using Python (version 3.5, https://www.python.org/).

Figure 1 compares series of national data over the period of 1960–2014, and the change characteristics of annual respiratory carbon emissions per unit area from human and livestock respiration could be analyzed for Afghanistan, South Korea, United Kingdom, China and United States (see Supplementary Table S2). The annual trend of human respiration is undoubtedly consistent with the trend of population growth in each countries. South Korea and China have remarkable upward trend, with 17.4–34.7 g C m^−2^ yr^−1^ for South Korea and 4.8–9.9 g C m^−2^ yr^−1^ for China, with averaged annual value of 28.0 g C m^−2^ yr^−1^ and 7.7 g C m^−2^ yr^−1^, respectively. Meanwhile, the human respiration of Afghanistan, United Kingdom and United States have little change with a higher averaged value of 16.3 g C m^−2^ yr^−1^ in United Kingdom. The variation of livestock respiratory carbon emissions fluctuated greatly, especially South Korea shows a remarkable increase after 1975. The livestock respiration in South Korean and United Kingdom is relatively greater, with averaged annual value of 32.9 g C m^−2^ yr^−1^ and 58.5 g C m^−2^ yr^−1^. Here, we note that the total livestock respiratory carbon emissions of South Korea is not large comparing with that of other countries, but the carbon emissions per unit area is high due to the small land area, which is similar to that of United Kingdom.

**Figure 2:**
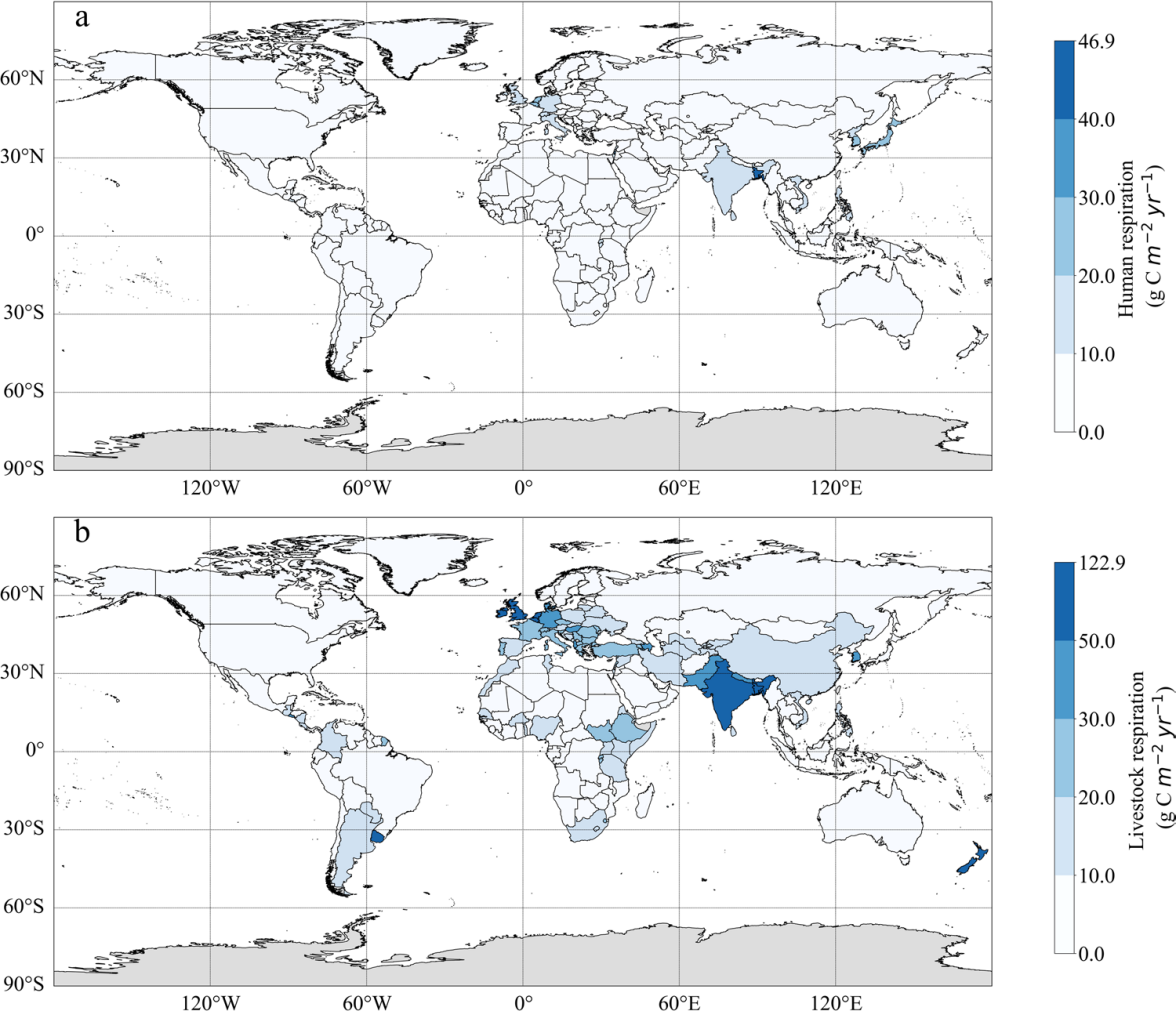
Averaged annual carbon emission (g C m^−2^ yr^−1^) from heterotrophic respiration of **(a)** human beings and **(b)** livestock during 1960–2014 in each country. The figure was generated using Python (version 3.5, https://www.python.org/) including packages: Matplotlib (http://matplotlib.org/)^38^ and geonamescache (https://pypi.python.org/pypi/geonamescache/), and the shapefile with country borders is based on the 1:10 m cultural vector from Natural Earth: (http://www.Naturalearthdata.com/).

We do similar analysis on 157 countries in the world. In most countries, the averaged annual value of carbon emissions from human is between 0.01–10 g C m^−2^ yr^−1^, with the largest value of 46.9 g C m^−2^ yr^−1^ in Bangladesh (Fig. 2a). As for the livestock, carbon emissions of almost all the countries is little than 70 g C m^−2^ yr^−1^, and the countries with the large values are mainly distributed in Europe, Asian and some countries of Africa and South America. The largest averaged annual livestock respiration are found with value of 122.9 g C m^−2^ yr^−1^, 67.2 g C m^−2^ yr^−1^, 65.9 g C m^−2^ yr^−1^ in Bangladesh, New Zealand and Netherlands, respectively (Fig. 2b).

## Discussion

**Figure 3:**
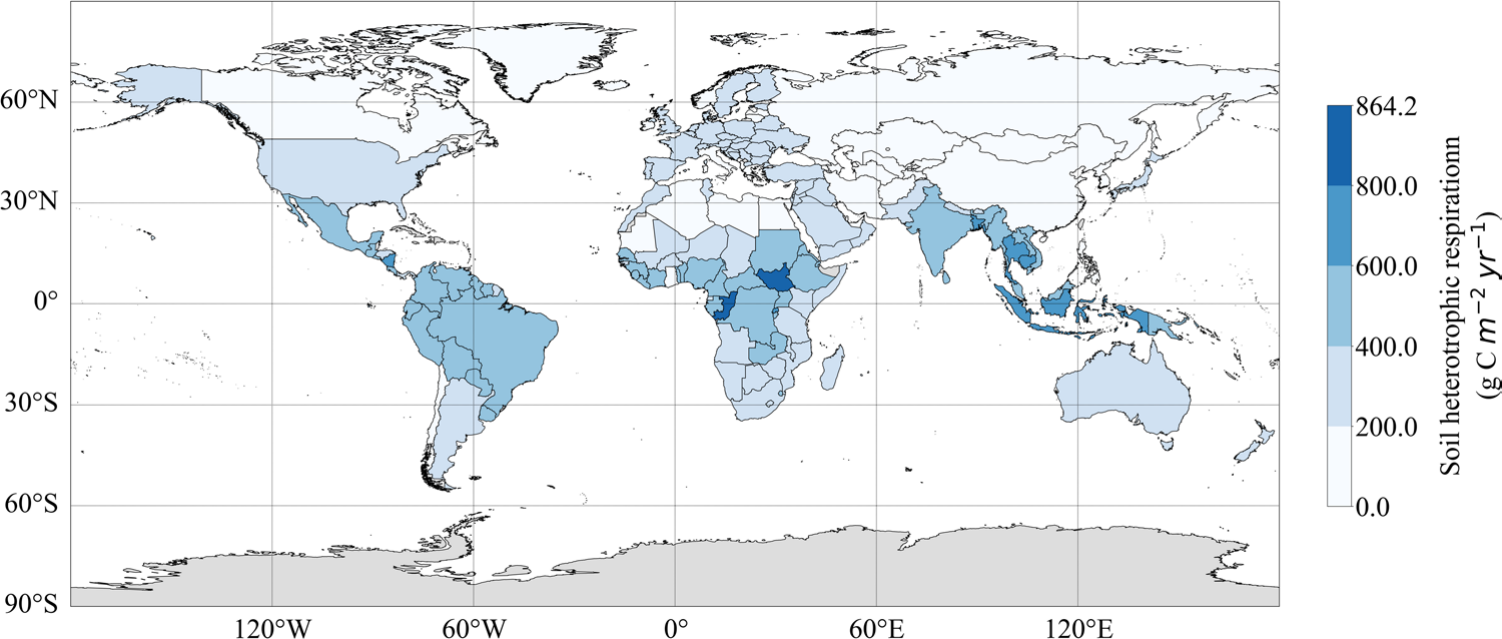
Distribution map of simulated global R_h_ in each country (kg C m^−2^ yr^−1^). The figure was generated using Python (version 3.5, https://www.python.org/) including packages: Matplotlib (http://matplotlib.org/)^38^ and geonamescache (https://pypi.python.org/pypi/geonamescache/), and the shapefile with country borders is based on the 1:10 m cultural vector from Natural Earth: (http://www.Naturalearthdata.com/).

It is suggested that the carbon output of urban ecosystem should include plant, soil and live creatures^23^. Expanding a single urban system to its surrounding areas even to a national scale, whether the study of human and livestock respiration is still making sense is the focuses in the following discussion. Trying to figure it out, this study estimates soil heterotrophic respiration (R_h_) as the heterotrophic respiration in terrestrial ecosystem, and the total heterotrophic respiration in each country is calculated as the sum of R_h_, human respiration and livestock respiration. Studies on estimating R_h_ in various regional scales mainly using model analysis^24–29^. Based on the measured data of R_h_ and soil database, we calculated the carbon emissions density from soil heterotrophic respiration averaged in each country within the range of 2.0–864.2 g C m^−2^ yr^−1^ (see Fig. 3 and Methods).

**Figure 4:**
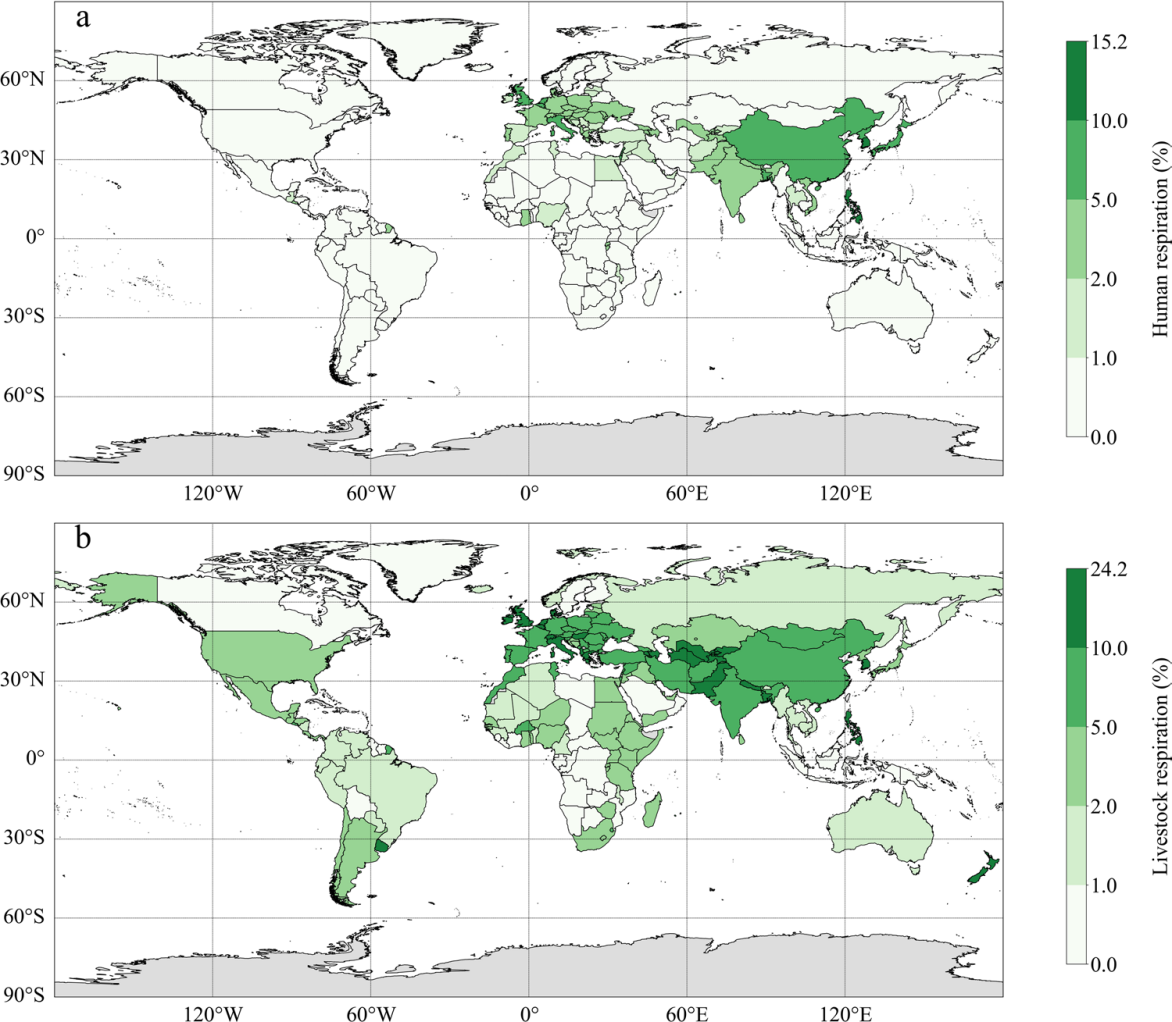
Averaged annual proportion (%) of each part of **(a)** human respiration and **(b)** livestock respiration during 1960–2014 in each country. The figure was generated using Python (version 3.5, https://www.python.org/) including packages: Matplotlib (http://matplotlib.org/)^38^ and geonamescache (https://pypi.python.org/pypi/geonamescache/), and the shapefile with country borders is based on the 1:10 m cultural vector from Natural Earth: (http://www.Naturalearthdata.com/).

Although the proportion of R_h_ is larger than 90% in most countries around the world, Fig. 4 showed greater contribution of human respiration to the total heterotrophic respiration in some developed countries in Europe, as well as some densely populated countries in Asia. Philippines and South Korea have the largest proportion of human respiration during 1960–2014 (respectively 15.2% and 11.9%). The proportion of livestock respiration has similar distribution to that of human. In some countries in Americas and Africa, the proportion of livestock respiration is greater than that of human within a range between 1% and 5%, and the greatest proportion is 24.2% in Ireland followed by 23.6% in New Zealand and 21.4% in Luxembourg. The number of countries in which the carbon emissions from human and livestock account for more than 1% of total heterotrophic respiration are respectively 74 and 127, making up 47.1% and 80.9% of the total studied countries. And the number of countries whose proportion is greater than 10% are 2 and 24, respectively. Furthermore, for the vast majority of countries, breathing activity of living creatures can contribute more than 1% of the total heterotrophic respiration (See Table 2).

**Table 2:** The number of regions with different heterotrophic respiration proportion.

The region of data	The proportion of total heterotrophic respiration	Human respiration		Livestock respiration	
		Number of counties	Percentage (%)	Number of counties	Percentage (%)
Countries around the world	>1%*	74	47.1	127	80.9
	1–5%	60	38.2	68	43.3
	5–10%	12	7.6	35	22.3
	>10%*	2	1.2	24	15.3

*Total number of countries with the proportion larger than given percentage.

Through the previous analysis, we argue that when estimating the carbon output in previous mentioned areas, unless the research object is a single ecosystem, such as forest, farmland, grassland or wetland (with relatively few people and animals), carbon emissions from human and livestock should be included in the calculations. Furthermore, the breeding of livestock is closely related to human activities, thus the absolute quantity of livestock respiration is consistent with human respiration in term of spatial distribution. Under this condition, our result also support that the composition and distribution of population is one of the potential drivers for the impact of urbanization on regional carbon budget^30^.

Although the effect of human and livestock is not very remarkable in some countries, more attention should be paid to the importance of heterotrophic respiration from aboveground creature. The wild animals such as those in Africa, as well as birds and insects which are difficult to take statistics will increase the total regional heterotrophic respiration. Otherwise, the actual carbon release from breathing would be greater than the parameters derived from BMR considering the temperature and physical activities. Therefore, the influence of human and animals respiration should be theoretically greater.

Due to the extremely small amount compared with R_h_, the possibility of double counting, as well as the insufficiency of the data at present, ordure of human and livestock, meat consumption, and death toll are not considered in this paper, although they indeed contribute to the carbon efflux of terrestrial ecosystems. In addition, since the period of collected data is limited, the interannual variability of human and animals respiration cannot be reflected in a longer time scale. In future studies, the time section would be extended, and the variation characteristics of human and livestock respiration in decades or even centuries under the background of global warming will be analyzed.

## Methods

### Data used to estimate human and livestock respiration

The land area and annual population (1960–2014) of the countries are downloaded from the World Bank website (The World Bank: http://data.worldbank.org/indicator)^31,32^. Livestock production of each country during 1960–2014 come from the Food and Agriculture Organization Corporate Statistical Database^33^. Because of the coverage area and the resolution of soil data, soil heterotrophic respiration is difficult to estimate for some small land countries and islands. Therefore, the study has to do a selection from the world’s 247 countries and regions (according to the cultural vector from Natural Earth: http://www.Naturalearthdata.com/). At the same time, some countries and regions do not have statistics data in the databases of FAOSTAT and the World Bank. Thus, we screens out 157 countries from the global countries for statistics and calculation. Moreover, countries with incomplete years are replaced by mean values of other years.

Considering the hardness of collecting for original data, the animals included in this study are only livestock, excluding zoo animals, wild animals, pets, insects, birds and so on, although they have a large quantity.

### Soil data

The soil parameters are consist of measured soil heterotrophic respiration (R_h_), soil organic matter, soil temperature and soil moisture. A Global Database of Soil Respiration Data (SRDB, Version 3.0) from the Oak Ridge National Laboratory (ORNL) collects 1487 studies on soil respiration from 1961 to 2011, where established 5173 records^34^. We obtained 254 records on R_h_ from the database, which derived from 183 studies, and were completed in 128 sites (see Supplementary Dataset 1). These records published during 1962 to 2010, and the distribution of data covers 34 countries around the world. In this paper, we used the soil carbon density (kg C m^−2^ yr^−1^) data from the Global Gridded Surfaces of Selected Soil Characteristics data published by IGBP-DIS at a resolution of 5 × 5 arc-minutes^35^. The global soil temperature (K) and soil moisture (%Vol.) data T62 Gaussian grid with 192 × 94 points were obtained from the National Centers for Environmental Prediction/National Center for Atmospheric Research (NCEP/NCAR) reanalysis project^36^. The dataset is in long-term monthly means and derived from data for the period of 1981–2010. For the following study, soil temperature and soil moisture at the depth of 0–100 cm was computed primarily and all the data was resampled into a global resolution of 0.5° × 0.5° using ArcGIS 10.2.

### R_h_ estimation equation

According to the empirical model of soil respiration and the function of R_h_ in a process-based modeling approach to simulate the soil production of CO_2_^37^, we constructed a model to calculate global R_h_ using the measured soil heterotrophic respiration data. Soil temperature, soil moisture and soil-carbon density are used as the main influencing factors in this study. The formulae used in this study is list as follows:

$${R}_{h}=OC\times {e}^{(-{k}_{1}\times f(T)\times f(w)+{k}_{2})}$$

$$f(T)=\frac{E}{R}\frac{T-10}{T\times 10}$$

$$f(W)=1-{e}^{(-a\times w+c)}$$

where R_h_ represents the soil heterotrophic respiration rates; OC is soil carbon density (kg C m^−2^ yr^−1^);  *f*(*T*) and  *f*(*W*) are scaling factors reflecting the dependence of R_h_ on soil temperature and soil moisture; E is the activation energy in kJ mol^−1^; R is the universal gas constant; T is the soil temperature in K; soil moisture W (% Val.) has two constant parameters as a = 15 and c = 0.11, respectively. In order to obtain the parameters k_1_ and k_2_ we need in the above formulae, soil temperature, soil moisture and soil carbon density are respectively matched with the latitude and the longitude of measured soil heterotrophic respiration data in SRDB database (see Supplementary Figs S1 and S2). Then we fitted measured R_h_ with soil temperature, soil moisture and carbon density values in applying the preceding formulae. The fitting formula of R_h_ is finally established with the parameters in the equation as k_1_ = −0.6283, k_2_ = 3.7878 (R = −0.69, P < 0.001).

The R_h_ was first simulated as grid data (see Supplementary Fig. S3), then we calculated the averaged carbon emissions density from R_h_ in each country using ArcGIS 10.2.

### Statistical analysis

All statistical analyses reported here were performed using MATLAB R2014a.

### Data availability

The datasets generated during the current study are available in Supplementary Dataset 2.

## Electronic supplementary material

Supplementary information

Dataset 1

Dataset 2
