# Supplementary material for: Global patterns of human and livestock respiration
Source: Sci Rep. 2018 Jun 18;8:9278. doi: 10.1038/s41598-018-27631-7 (PMC6006152; doi:10.1038/s41598-018-27631-7)
Supplement: Supplementary file 1 — Supplementary information [file 41598_2018_27631_MOESM1_ESM.pdf]

Global patterns of human and livestock respiration

Qixiang Cai, Xiaodong Yan*, Yafei Li, Leibin Wang
correspondence to: yxd@bnu.edu.cn.

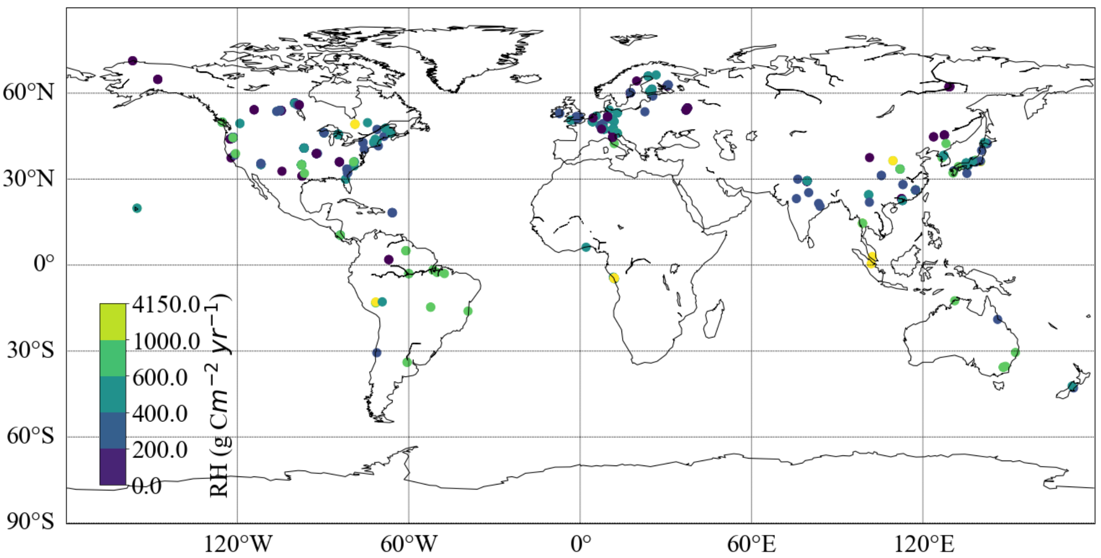

Supplementary Figure S1. The distribution of 254 published data of R_h (kg C m^{-2}) used in this study. The figure was generated using Python (version 3.5, https://www.python.org/) including packages: Matplotlib (http://matplotlib.org/) [38]

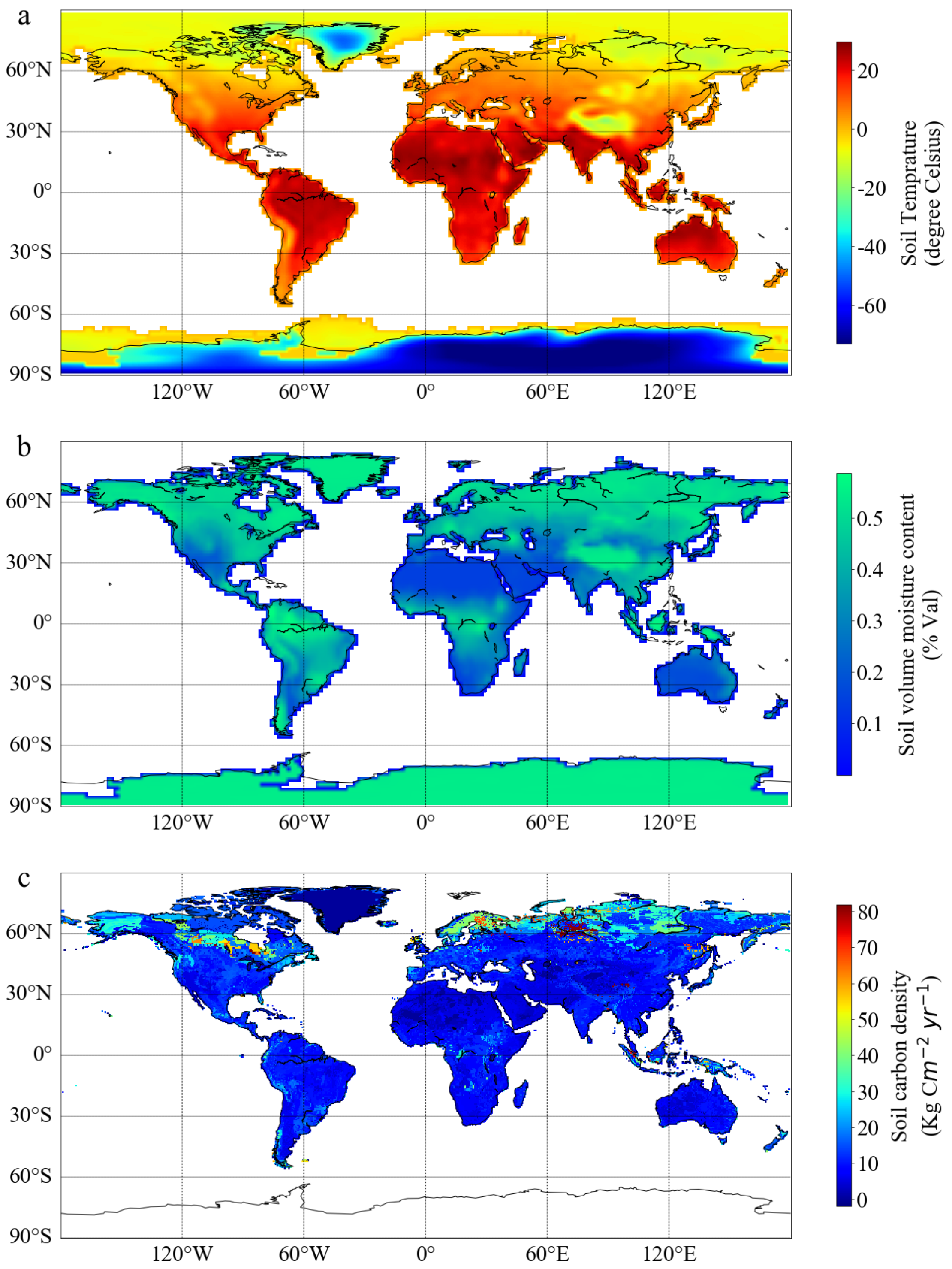

Supplementary Figure S2. Global spatial distribution of **A,** soil temperature (K); **B,** soil volume moisture content (% Val.); **C,** soil carbon density (kg C m^{-2} yr^{-1}) at a grid resolution of 0.5°×0.5°. The figure was generated using Python (version 3.5, https://www.python.org/) including packages: Matplotlib (http://matplotlib.org/) [38]

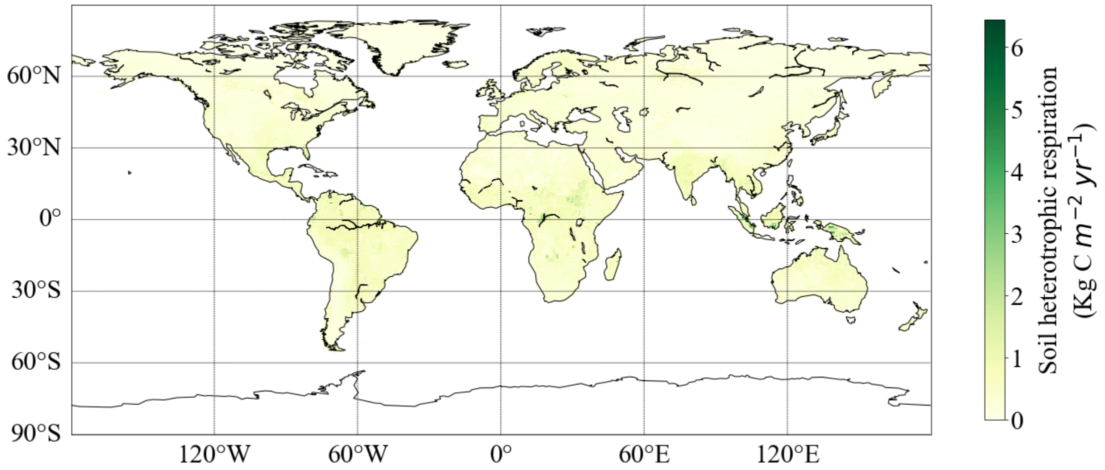

Supplementary Figure S3. Distribution map of the simulated global soil heterotrophic respiration R_h (kg C m^{-2} yr^{-1}). The figure was generated using Python (version 3.5, https://www.python.org/) including packages: Matplotlib (http://matplotlib.org/) [38]

Taxon	Body weight(g)	BMR(ml O_2 g^{-1} h^{-1})	BMR (Kg O_2 y^{-1})
Equus caballus	260000	0.25	813.429
Sus scrofa	75000	0.11	103.243
Camelus dromedarius	407000	0.1	509.331
Bos taurus	272000	0.17	578.661
Capra sp.	36000	0.19	85.5977
Ovis aries	30000	0.34	127.646
broilers	862.3	2.497601	26.95179
Rabbits	3000	0.5	18.77143
rat	400	0.8	4.004571
Human being	70000	0.21	183.96

*We converted the oxygen consumed by respiration into carbon according to the equation as follow:

$$C_6H_{12}O_6+6O_2+6H_2O \rightarrow 6CO_2+12H_2O$$

where $C_6H_{12}O_6$ represents the Glucose. We only considered the process of aerobic respiration.

Supplementary Table S1. Original data of basal metabolic rate (BMR) used for the calculation of carbon emissions from human and livestock*

country	Total heterotrophic respiration (g C m^{-2} yr^{-1})	Soil		Human		Livestock	
		Value (g C m^{-2} yr^{-1})	Proportion (%)	Value (g C m^{-2} yr^{-1})	Proportion (%)	Value (g C m^{-2} yr^{-1})	Proportion (%)
Afghanistan	157.4	145.9	0.93	1.7	0.01	9.8	0.06
South Korea	234.5	173.7	0.74	28.0	0.12	32.9	0.14
United Kingdom	279.5	204.7	0.73	16.3	0.06	58.5	0.21
China	148.2	128.9	0.87	7.7	0.05	11.6	0.08
United States	221.4	213.3	0.96	1.8	0.01	6.3	0.03

Supplementary Table S2. The proportion of heterotrophic respiration components in each sample country
